# Knee strength balance ratios are not affected by aging among male runners

**DOI:** 10.1016/j.jesf.2025.01.003

**Published:** 2025-01-11

**Authors:** Ronaldo Alves da Cunha, Vinícius Ribeiro dos Anjos Souza, Lavínia Vivan, Aldo Seffrin, Claudio Andre Barbosa de Lira, Katja Weiss, Beat Knechtle, Marilia Santos Andrade

**Affiliations:** a Postgraduate Program in Translation Medicine, Federal University of São Paulo, São Paulo, São Paulo, Brazil; b Human and Exercise Physiology Division, Faculty of Physical Education and Dance, Federal University of Goiás, Goiânia, Goiás, Brazil; c Institute of Primary Care, University of Zurich, Zurich, Switzerland; d Medbase St. Gallen Am Vadianplatz, St. Gallen, Switzerland; e Department of Physiology, Federal University of São Paulo, São Paulo, São Paulo, Brazil

**Keywords:** Strength, Isokinetic, Aging, Balance ratio

## Abstract

**Objectives:**

This study aimed to assess thigh muscular strength, conventional and functional strength balance ratios, and muscle quality in well-trained male runners aged 20–70 yr.

**Methods:**

Eighty-nine male runners were divided into five age categories: 20–29, 30–39, 40–49, 50–59, and 60–70 yr. Participants underwent an isokinetic strength test for knee flexor and extensor muscles and a body composition analysis via dual-energy X-ray absorptiometry.

**Results:**

No significant difference was observed in concentric peak torque of the knee extensor muscles between the 20–29 and 40–49 age groups. However, the 50–59 age group showed significantly lower strength than the 20–29 age group (*p* = 0.049), and the 60–70 age group demonstrated significantly lower strength than the 40–49 group (*p* < 0.001). The conventional and functional balance ratios remain stable across all age groups.

**Conclusion:**

Knee flexor and extensor strength in male runners decreases significantly after the age of 50, while conventional and functional strength balance ratios remain stable.

## Statements relating to our ethics and integrity policies

The study was approved by the Institutional Ethics Committee of the Federal University of São Paulo, Brazil (approval number 4.354.386). Moreover, participants signed a consent form after being provided with information regarding the study's objectives, experimental procedures, risks, and benefits, as well as the assurance of privacy and confidentiality.

## 1 Introduction

Running improves aerobic capacity and skeletal muscle aerobic status, yet its effects on muscle strength remain uncertain despite the crucial role of strength in athletic performance, daily activities, and overall survival.^1^, ^2^, ^3^ It is well established that skeletal muscle mass begins to decline in the third decade of life,^3^ with approximately 10 % of muscle mass lost by age 50. The decline accelerates after the age of 70 yr, with muscle strength decreasing at an estimated rate of 3.5 % per year.^4^ The decline in muscle strength often exceeds that of muscle mass, indicating a deterioration in muscle quality.

Investigating whether running mitigates the age-related decline in muscle strength is important, as it could help extend the number of years individuals live independently.^1^ However, running-related musculoskeletal injuries (RRMI) are common, despite the health benefits of the sport.^5^ These injuries often result from muscular weaknesses, bilateral asymmetries, or imbalances between antagonist and agonist muscles, which increase the risk of injury.^6^, ^7^, ^8^ For example, muscular asymmetry has been associated with higher knee loads during weight acceptance while running, elevating the risk of knee injuries.^9^ Additionally, imbalances in muscle strength between the knee flexor and extensor muscles are linked to injuries, with hamstring strains being one of the most common injuries among runners.^10^, ^11^, ^12^ By recognizing these risk factors, targeted strengthening programs can be implemented to reduce the incidence of RRMI. Therefore, examining the strength profiles of the knee flexor and extensor muscles in various age groups is essential because of the significance of muscular strength, symmetry, and balance in injury prevention and general health.

Understanding these risk variables is crucial to developing focused strengthening programs that lower the prevalence of RRMI. Examining the strength profiles of the knee flexor and extensor muscles in runners of various ages is essential because of the significance of muscular symmetry, and balance between for the avoidance of injuries and general health. Strength and conditioning specialists might use this information to create efficient programs and set realistic training objectives.

This study examined thigh muscle function, strength balance ratios and muscular symmetry across different age groups of male recreational runners. We hypothesize that strength losses would occur proportionately in both knee flexor and extensor muscles, resulting in stable strength ratios.

## 2 Methods

### 2.1 Ethical approval

All experimental procedures were reviewed and approved by the Human Research Ethics Committee of the Federal University of São Paulo (approval number 4.354.386, October 22, 2020) and conformed to the principles outlined in the Declaration of Helsinki. Moreover, participants signed an informed consent form after receiving information about the study's objectives, experimental procedures, risks, and benefits, and assurances of privacy and confidentiality.

### 2.2 Study design

This cross-sectional study, conducted between November 2019 and March 2020, involved 89 male runners aged 20–70 yr. The researchers contacted running coaches and invited runners who were training under their supervision to participate in the study. Athletes who were interested contacted the researchers via email or phone to participate, provided they met the inclusion criteria and did not meet any exclusion criteria.

Eligibility criteria required participants to engage in regular running, defined as completing at least three training sessions per week, for a minimum of 3 yr.

All participants underwent muscle function evaluation using an isokinetic dynamometer and body composition analysis via dual-energy X-ray absorptiometry (DXA), which controls movement speed to ensure constant velocity during testing.^13^ The runners were categorized into five age groups: 20–29, 30–39, 40–49, 50–59, and 60–70 yr.

Comparative analyses of knee muscle strength and balance ratios were performed across these age groups to investigate age-related changes in muscle performance and neuromuscular balance.

### 2.3 Participants

In this study, a convenience sample was used. People who ran were encouraged to join by handing out flier cards in running communities and on social media. A total of 89 male runners were recruited. The age and anthropometric details of participants are shown in Table 1. Table 2 contains information on the number of training sessions per week, the chosen race distances, the years of running experience, the availability of coach counseling, and the primary driving force behind running.

**Table 1:** Anthropometric and age characteristics of runners in this study (n = 89).

Characteristics	Age groups (years)					*p value*
	20-29 (n = 10)	30-39 (n = 21)	40-49 (n = 23)	50-59 (n = 22)	60 -70 (n = 13)	
Age (years)	27.4 (1.4)^a^	35.7 (2.3)^a^	44.1 (3.03)^a^	53.4 (2.3)^a^	66.2 (3.8)^a^	<0.001
Height (m)	1.73 (0.06)	1.77 (0.06)	1.75 (0.08)	1.74 (0.07)	1.73 (0.08)	0.873
Body mass (kg)	73.8 (7.0)	75.2 (7.9)	77.4 (11.7)	74.5(11.5)	75.6(12.1)	0.342
BMI (kg/m^2^)	24.5 (1.5)	23.8 (2.6)	25.1 (3.0)	24.5 (3.1)	24.9 (2.3)	0.672

Data are expressed as mean and standard deviation (tested by the one-way ANOVA test). BMI: body mass index.

a *p* < 0.05 (different from all others age groups).

**Table 2:** Characteristics of runners in this study (n = 89).

	20-29 (n = 10)	30-39 (n = 21)	40-49 (n = 23)	50-59 (n = 22)	60 -70 (n = 13)	
Education status, n (%)						0.323
Elementary school	–	–	2 (8.7)	–	–	
High school	5 (50)	7 (33.3)	6 (26.1)	7 (31.8)	2 (15.4)	
Graduate	5 (50)	14 (66.7)	15 (65.2)	15 (68.2)	11 (84.6)	
Running experience (years)	2 (1.4–10)∗^&^	6 (1–10) ∗^&^	7 (3–24) ∗^&^	15 (3–35)	19 (5–40)	<0.001
Training sessions/week	3 (1.87)	3.4 (1.66)	2.94 (1.5)	3 (1.6)	3.3 (1.2)	0.926
Km per week	55.4 (30.3)∗	40.7 (25.6)	33.5 (16.1)	44 (15.8)	27.7 (19.5)	0.039
Preferred races, n (%)						0.343
<10 km	3 (30)	4 (19)	2 (8.7)	5 (22.7)	2 (15.4)	
10 km	2 (20)	8 (38.1)	11(47.8)	4 (18.2)	2 (15.4)	
>10 km and ≤21.5 km	5 (50)	7 (33.3)	8 (34.8)	13 (59.1)	7 (53.8)	
Marathon	0	2 (9.5)	2 (8.7)	0	1 (7.7)	
Ultramarathon	0	0	0	0	1 (7.7)	
Coach, n (%)						0.458
Yes	7 (70)	13 (61.9)	12 (52.2)	9 (40.9)	6 (46.2)	
No	3 (30)	8 (38.1)	11 (47.8)	13 (59.1)	7 (53.8)	
Main motivation to run, n (%)						0.094
Health	2 (20)	6 (28.6)	6 (26.1)	5 (22.7)	9 (69.2)	
Performance	2 (20)	6 (28.6)	4 (17.4)	2 (9.1)	0	
Aesthetics	1 (10)	0	0	1(4.5)	0	
Social	5 (50)	9 (42.9)	13 (56.5)	14 (63.6)	4(30.8)	

Continuous data are expressed as mean and standard deviation (tested by the one-way ANOVA test). All categorical data are expressed by percentages and number of participants (tested by the Chi-square test). ∗*p* < 0.05 (different from ≥60 years old); ^&^*p* < 0.05 (different from 50 to 59 years old).

Male runners who were 20 yr of age or older and fulfilled the following requirements were eligible to participate. They had to have who completed the baseline questionnaire and reported running regularly (at least three times per week, as most runners tend to do) for at least the previous 3 yr.^14^^,^^15^ Runners with conditions prohibiting them from participating in the isokinetic or body composition tests, limitations on their ability to exert maximum effort, or lower limb edema or pain were not allowed to continue. Women were not included in this study since the purpose was to assess the impact of aging on muscle performance rather than the impacts of the menstrual cycle, and because female strength may be impacted by this phase.^16^ We acknowledge, however, that the underrepresentation of women in science hinders diversity and equity. Future research on the impact of aging on muscle performance should address this issue to ensure more inclusive and representative findings.

### 2.4 Data collection

Two stages were used in the data collection process. In the first stage, participants completed a paper-based questionnaire on their demographic characteristics, running experience, training routines, participation in other sports, and current medical status. The second stage involved a body composition evaluation, followed by an isokinetic muscle strength assessment. All assessments were conducted between 8 a.m. and 12 p.m. to minimize the effects of circadian rhythms on muscular function.^16^ All data were collected by a professional with over 3 yr of experience.

### 2.5 Isokinetic muscle evaluation

Isokinetic strength was assessed using a Biodex System 4® dynamometer (Biodex Medical System, Shirley, New York, USA). Before testing, all participants performed a standardized warm-up consisting of 5 min of stair climbing at a light intensity, as perceived by the volunteer, followed by dynamic stretching to prevent stretching-induced effects on strength measurements.^17^ The participant was instructed to stand on one leg and perform 10 large, continuous hip flexion and extension movements, followed by 10 hip adduction and abduction movements with the non-weight-bearing lower limb. Subsequently, the support leg was switched, and the exercise was repeated for the other lower limb. Participants were informed about the evaluation process, visual feedback provided during testing, and the protocol for stopping the test in case of an emergency.

During testing, participants received consistent verbal encouragement to explore maximum effort. Each participant was seated with an 85° hip flexion angle, and their torso, hips, and thighs were secured with straps to minimize extraneous movement and isolate the knee joint. The distal attachment was placed 2 cm above the lateral malleolus, with the knee joint aligned with the lateral femoral condyle at 90° of flexion. The knee range of motion was restricted to 0°–90°. To ensure precision, gravity was compensated for using the system software.^18^

The testing protocol consisted of five maximum repetitions at regular velocities of 60°/s and 180°/s in concentric mode and 60°/s in eccentric mode. For validity, the coefficient of variation across the five repetitions had to remain below 5 %.^18^ All tests met this criterion. To familiarize participants with the equipment and resistance, three submaximal extension/flexion trials were conducted before testing.^8^

The evaluated variables included concentric (conc) and eccentric (ecc) peak torque (PT) values in Newton-meters (Nm). The conventional balance ratio was calculated as flexor muscles conc PT/extensor muscles conc PT at 60°/s.^19^ The functional balance ratio was calculated as flexor muscles ecc PT/extensor muscles conc PT at 60°/s.^20^ Muscular symmetry was calculated as (extensor muscles PT of dominant limb - extensor muscles PT of nondominant limb)/extensor muscles PT of dominant limb × 100.^19^ The same ratio was calculated for flexor muscles. Muscle quality was measured as the sum of extensor and flexor PT muscles divided by lower limb lean mass (kg) measured using DXA.

### 2.6 Body composition evaluation

Dual-energy X-ray absorptiometry was used to measure body composition, with participants lying supine on the equipment table. Calibration was performed before each scan according to the manufacturer's guidelines.^21^ This technique is widely accepted for its reliability and low radiation exposure. Lower limb lean mass (kg) was recorded and used to calculate muscle quality. Participants wore comfortable clothing free of metal components, and all DXA scans were conducted in a private room by the same experienced evaluator.

### 2.7 Data analysis

The required sample size was calculated based on an effect size of 0.40, a significance level of 0.05, and a power of 0.80. The results indicated that 80 participants (16 per group) were required. Sample size and power calculations were performed using G∗Power software (version 3.1.9.7, Heinrich-Heine-Universität Düsseldorf, Düsseldorf, Germany). Data analysis was conducted using Microsoft® Excel® 2020 (version 2013) and R software (version 1.4.1106, R Foundation for Statistical Computing, Vienna, Austria).

Descriptive statistics was used to summarize the sample characteristics. Normally distributed continuous variables were reported as mean and standard deviation, whereas categorical variables were expressed as frequencies (*n*) and percentages. Histograms and probability density functions were used to assess normality.

A one-way ANOVA was used to evaluate group differences in isokinetic parameters. Tukey's *post hoc* tests were applied to identify specific differences when necessary. If no significant interactions were found, only main effects were reported. Statistical significance was set at *p* < 0.05.

## 3 Results

Body mass index, educational level, weekly training sessions, race preferences, coaching status, running motivation, and self-assessment were comparable across age groups. However, the weekly distance run was shorter in the 60–70-year-old group than in the 20–29-year-old group (*p* < 0.05; Table 1, Table 2).

The 60–70-year-old group had significantly lower concentric PT at 60°·s^−1^ in knee extensors than the 20–29-year-old group (mean difference = 61.6; 95 % CI, 41.1–82.1, *p* < 0.001), 30–39-year-old group (mean difference = 50.3; 95 % CI, 35.2–65.4, *p* < 0.001), and 40–49-year-old group (mean difference = 46.6; 95 % CI, 23.6–69.6, *p* < 0.001). Furthermore, the 50–59-year-old group produced considerably less concentric PT than the 20–29-year-old group (mean difference = 33.2, 95 % CI = 6.56–59.84, *p* = 0.049). Similar differences were observed for concentric PT at 180°·s^−1^ in knee extensors, as the values were significantly lower in the 60–70-year-old group than the 20–29-year-old group (mean difference = 41.6; 95 % CI, 26.8–56.4, *p* < 0.001) and the 30–39-year-old group (mean difference = 36.5; 95 % CI, 23.5–49.5, *p* < 0.001). Additionally, the 60–70-year-old group had significantly reduced muscle strength than the 40–49-year-old group (mean difference = 28.8; 95 % CI, 13.3–44.3, *p* = 0.003) and the 50-59-year-old group (mean difference = 22.5; 95 % CI, 9.5–35.5 *p* = 0.034). However, there were no significant differences in eccentric PT of the knee extensor muscles at 60°·s^−1^ among age groups (Table 3).

**Table 3:** Isokinetic muscle evaluation of dominant knee extensor and flexor muscles of age groups.

	Age groups					p– value	power
	20–29 (n = 10)	30–39 (n = 21)	40–49 (n = 23)	50–59 (n = 22)	60–70 (n = 13)		
**Knee extensor muscles**							
Concentric 60°.s−1							
peak torque (Nm)	206.6 (29.3)∗∗§	195.3 (22.7)∗∗	191.6 (38.4)∗∗	173.4 (36.1)	145.0 (17.7)	<0.001	0.95
Concentric 180°.s−1							
peak torque (Nm)	143.9 (19.5)∗∗	138.8 (19.8)∗∗	131.1 (25.1)∗	124.8 (24.5)∗	102.3 (14.7)	<0.001	0.96
Eccentric 60°.s−1							
peak torque (Nm)	231.2 (76.4)	243.0 (53.0)	257.2 (58.4)	228.1 (48.5)	215.6 (39.5)	0.406	0.72
**Knee flexor muscles**							
Concentric 60°.s−1							
peak torque (Nm)	111.0 (20.1)∗∗§	101.0 (21.2)∗	103.0 (20.9)∗∗	88.9 (19.0)	75.9 (9.9)	<0.001	0.84
Concentric 180°.s−1							
peak torque (Nm)	78.5 (18.4)∗	75.0 (17.6)∗	77.0 (15.2)∗∗	65.3 (12.0)	55.0 (11.0)	<0.001	0.52
Eccentric 60°.s−1							
peak torque (Nm)	178.4 (22.1)∗	167.1 (36.1)	169.9 (39.9)	152.8 (26.1)	140.6 (22.1)	0.024	0.97
**Muscle quality (Nm/kg)**	15.7 (0.9)	16.4 (1.8)∗	16.1 (2.9)∗	15.7 (2.3)	13.0 (2.9)	0.013	0.98

Data are expressed as mean and standard deviation. §p < 0.05 (different from 50 to 59 years old); ∗∗p < 0.001 (different from ≥60 years old); ∗p < 0.05 (different from ≥60 to 70 years old).

The concentric PT at 60°·s^−1^ in knee flexors was significantly lower in the 60–70-year-old group than the 20–29-year-old group (mean difference = 35.1; 95 % CI, 21.9–48.3, *p* < 0.001), 30–39-year-old group (mean difference = 25.1; 95 % CI, 12.3–37.9, *p* = 0.003), and 40–49-year-old group (mean difference = 27.1; 95 % CI, 14.5–39.7, *p* < 0.001). The 50–59-year-old group also exhibited significantly lower muscle strength than the 20–29-year-old group (mean difference = 22.2; 95 % CI, 3.9–40.2, *p* = 0.027). The concentric PT at 180°·s^−1^ in knee flexors was significantly lower in 60–70-year-old group than the 20–29-year-old group (mean difference = 23.5; 95 % CI, 10.7–36.3, *p* = 0.003), 30–39-year-old group (mean difference = 20.1; 95 % CI, 8.9–31.1, *p* = 0.002), 40–49-year-old group (mean difference = 22.0; 95 % CI, 12.22–31.8, *p* < 0.001).

Table 3 shows that the eccentric PT of the knee flexor muscles at 60°·s^−1^ was significantly lower in the 60–70-year-old group than in the 20–29-year-old group (mean difference = 37.8; 95 % CI, 18.5–57.1, *p* = 0.047). Muscle quality in the 60–70-year-old group was also significantly worse than in the 30–39-year-old and 40–49-year-old groups (*p* < 0.001; Table 3). However, there were no significant changes among the groups in terms of knee extensor and flexor muscle symmetry as well as conventional and functional strength ratios (Table 4). The mean and standard deviation values for CR and FR are presented in Table 4. The confidence intervals (CIs) for CR are as follows: 20–29 group (46.9–58.7, 95 % CI), 30–39 group (47.4–55.5, 95 % CI), 40–49 group (49.5–57.2, 95 % CI), 50–59 group (49.2–57.1, 95 % CI), and 60–70 group (46.5–56.9, 95 % CI). Similarly, the CIs for FR are: 20–29 group (71.8–94.8, 95 % CI), 30–39 group (71.8–87.6, 95 % CI), 40–49 group (77.8–93.0, 95 % CI), 50–59 group (76.4–91.9, 95 % CI), and 60–70 group (79.5–99.7, 95 % CI).

**Table 4:** Limb symmetry indexes and knee flexor-extensor strength ratios (CR and FR) at 60°·s−1 of age groups.

	Age groups (years)						
	20–29 (n = 10)	30–39 (n = 21)	40–49 (n = 23)	50–59 (n = 22)	60–70 (n = 13)	*p*– value	*power*
Knee extensor muscles							
concentric 60°.s−1 (%)	9.5 (6.9)	10.5 (7.8)	9.4 (9.7)	12.1 (6.9)	8.1 (7.2)	0.416	0.74
concentric 180°.s−1 (%)	7.2 (4.2)	4.8 (4.1)	7.1 (4.9)	8.7 (4.9)	8.7 (6.6)	0.624	0.99
Knee flexor muscles							
concentric 60°.s−1 (%)	8.8 (4.7)	11.1 (7.6)	10.1 (8.0)	13.5 (10.8)	12.5 (6.7)	0.455	0.96
concentric 180°.s−1 (%)	8.5 (6.7)	10.8 (9.2)	11.9 (11.2)	10.5 (8.3)	7.4 (4.9)	0.997	0.99
CR 60°.s−1 (%)	53.9 (7.9)	51.5 (7.2)	53.9 (5.2)	52.7 (15.3)	52.7 (6.8)	0.953	0.97
FR 60°.s−1 (%)	83.3 (15.0)	79.7 (15.7)	85.4 (11.2)	84.2 (27.3)	89.6 (14.9)	0.387	0.72

Data are expressed as mean and standard deviation. CR: conventional balance ratio; FR: functional balance ratio.

## 4 Discussion

It is well-recognized that aging reduces muscle strength. However, it remains unclear whether this decline affects the knee flexor and extensor muscles equally. This issue is important, as any imbalance between these muscle groups could impact the knee strength ratio. To clarify this issue, we conducted a cross-sectional study involving 89 recreational male runners of various ages to assess the isokinetic strength of the knee extensor and flexor muscles. The study revealed the following findings: (a) knee flexor and extensor PT at 60°·s^⁻1^ was comparable between the 20–29 and 40–49 age groups, with significant strength reductions observed only after the age of 50; (b) muscle quality in the 60–70 age group was lower than in the 30–39 and 40–49 age groups; and (c) no significant differences were found in conventional and functional strength balance ratios across the age groups. Because the strength decline was comparable between the knee flexor and extensor muscles, the strength balance ratios remained consistent, supporting the initial hypothesis.

The 20–29, 30–39, and 40–49 age groups showed significant variations in knee flexor and flexor PT at 60°·s⁻^1^ in concentric mode. At 180°·s⁻^1^, however, no significant differences were found among these age groups. Only the 60–70 age group demonstrated a substantial reduction in muscle strength compared with the younger groups. Previous studies on nonathletes have reported a progressive decline in strength starting from age 30, with more pronounced reductions by age 50.^22^ These results suggest that muscle strength deterioration is a physiological consequence of aging,^23^ which can lead to functional limitations and impairments in daily activities.^24^ The loss of muscle strength that accompanies aging can be attributed to various factors. One of these factors may be an increased resistance to anabolic stimulation, such as muscle contraction, dietary amino acids, or anabolic hormones. This phenomenon, known as anabolic resistance, refers to a reduced ability to enhance protein synthesis rates and respond to anabolic stimuli. Another potential explanation for strength loss is the reduction in the size and number of type II muscle fibers, suggesting a shift from fast to slow fiber types^25^, ^26^, ^27^ Additionally, mitochondrial dysfunction, a well-recognized characteristic of aging, may also contribute to strength loss. A functional decline in mitochondrial quality and activity has been previously demonstrated, attributed to the destruction of mitochondrial structure and a reduction in mitochondrial content. Finally, another possible factor associated with strength loss during aging is the decline in cellular NAD + levels across most tissues, including skeletal muscle. NAD+ is essential for metabolic processes, as it accepts and donates electrons in glycolysis, β-oxidation, and the electron transport chain. Therefore, lower NAD + levels may negatively impact the ATP resynthesis process.It has been previously demonstrated a functional decline of mitochondrial quality and activity due to destruction of mitochondrial structure, and reduction of mitochondrial content.^28^ Finally, another possible factor associated with strength loss at aging is the cellular NAD + levels decline in the majority of tissues, including skeletal muscle. NAD+ is essential for metabolic processes as it accepts and donates electrons for glycolysis, β-oxidation, and the electron transport chain,^29^ therefore lower NAD + levels, may negatively impact the ATP resynthesis process.^30^

The findings of this study are relevant because they suggest that regular running can mitigate the adverse effects of aging on muscle strength. Significant reductions in muscle performance were only observed after the age of 50 or 60, implying that running may be an effective strategy to minimize age-related muscle strength loss. Health professionals should encourage regular running as part of a healthy lifestyle to preserve muscle function.

Previous research has also shown that, along with muscle strength loss, older adults experience reduced muscle force per unit area. For example, older rats demonstrated 34 % lower muscle quality (strength per unit of muscle mass) than younger rats.^31^ In this study, muscle quality (PT per unit of lean mass) was assessed, with lower values observed in the older group (60–70 yr) than in younger groups. These results are consistent with earlier studies tracking 1880 older adults over 3 yr, showing that strength declines faster than muscle mass, indicating a reduction in muscle quality.^3^^,^^20^

There were no significant differences in knee flexor and extensor muscle symmetry across the age groups, with mean asymmetry values below 15 %, which is considered within the normal range.^32^ Similar symmetry values, below 10 %, were reported in younger male long-distance runners (aged 18.0 ± 0.9 yr).^33^ These findings suggest that runners, regardless of age, do not exhibit muscle asymmetry as a risk factor for lower limb injuries.

The CR was measured at 60°·s⁻^1^ and 180°·s⁻^1^, with no significant differences between age groups, suggesting that both the quadriceps and hamstrings muscles undergo a comparable decline in muscle strength. The CR values ranged between 50 % and 60 %. A recent study reported slightly higher CR values (approximately 60 %) in well-trained younger male long-distance runners, potentially due to their younger age.^32^ The literature recommends a normative CR value of 60 % (assessed at 60°·s⁻^1^) as a reference point.^34^ A CR below 60 % indicates insufficient flexor muscle strength to counteract the stronger extensor muscles, increasing the risk of knee joint instability.^35^ As CR values in this study were consistently below 60 % across all age groups, strengthening the knee flexor muscles should be prioritized to improve CR. The low CR exhibited by runners can be attributed to the higher activation of the quadriceps muscle relative to the hamstrings during running at a majority of effort intensities, with the exception of VO2max intensity.^36^

This study also evaluated the FR, and, similar to the CR, no significant differences were found across the age groups, with FR values consistently below 1.0. Traditional guidelines suggest that FR values should exceed 1.0, as the eccentric antagonist torque should be greater than the concentric agonist torque to decelerate knee extension and maintain dynamic stability.^37^^,^^38^ Higher FR values (>1.0) have been reported in recreational runners when assessed at 240°·s⁻^1^.^39^^,^^40^ In this study, FR was assessed at 60°·s⁻^1^, and lower values were expected at this angular velocity. According to the force-velocity relationship, eccentric hamstring torque remains relatively constant, while concentric quadriceps torque increases at lower angular velocities.^41^ The FR values of <1.0 in this study are consistent with previous literature at 60°·s⁻^1^.^35^

One important observation is that before the study began, we calculated the required sample size for an alpha level of 0.05 and a power of 80 %. Our calculations indicated that we needed 16 participants in each age group. The 20–29 years old and the 60–70 years old groups had slightly smaller sample sizes, which could have compromised the ability to detect a significant effect, increasing the error type II. However, the post-hoc power analysis revealed that the power was greater than 80 %, suggesting that our sample size was adequate to produce reliable results and type II error lower than 20 %.

A limitation of this study is the absence of a control group, which would have allowed for a comparison of age-related strength changes between runners and nonathletes validating the protective effects of running training on knee strength. Additionally, the exclusion of female participants is noteworthy, as the effects of aging on muscle strength may differ between genders. Future research should include control groups including runners of different genders and age groups to provide more comprehensive insights into the impact of aging on muscle strength.

## 5 Conclusion

This study demonstrates that muscle strength and quality decline with age, becoming more apparent after the age of 50. Notably, conventional strength ratios (CRs) stayed consistent across age groups, a key factor in joint stability, however, it was below the recommended 60 % across all age groups, indicating the need for runners of all ages to include knee flexor strengthening exercises into their routines to improve muscle balance and joint stability.

## Data availability statement

The data supporting this study's findings will be made available upon request.

## Funding

This research received no financial or material support.

## Conflict of interest disclosure

The authors declare no conflicts of interest relevant to this article.
